# Supplementary material for: Mortality risk prediction of high-sensitivity C-reactive protein in suspected acute coronary syndrome: A cohort study
Source: PLoS Med. 2022 Feb 22;19(2):e1003911. doi: 10.1371/journal.pmed.1003911 (PMC8863282; doi:10.1371/journal.pmed.1003911)
Supplement: S1 Analysis Plan — (DOCX) [file pmed.1003911.s003.docx]

**S1 Analysis Plan. Statistical analysis plan.**

#### Study population

The study dataset will include all patients who have had a troponin measured at each of the five academic centres between 2010 (2008 for University College Hospital) and 1^st^ April 2017.

Exclusion criteria:

- No CRP or white cell count (WCC) measured
- CRP>15 mg/L
- WCC < 4x10^9^/L or WCC > 11x10^9^/L

The study population will be focussing on those with normal WCC in order to exclude:

- overtly septic patients
- those with haematological disorders that may have a major effect on levels of CRP

We will exclude all patients with CRP >15mg/L in a separate effort to exclude those with clinically significant inflammation or infection.

#### Data variables

All analyses on CRP and other haematological and biochemical blood tests, apart from troponin, will be performed using the first result measured during the hospital care episode.

1. **CRP**

The 99^th^ percentile of the upper limit of normal (ULN) and the limit of detection of all CRP assays used across all cardiac centres will be determined.

The population will be divided into four categories according to CRP level:

1. normal CRP (<2mg/L)
2. CRP 2-4.9 mg/L
3. CRP 5-9.9 mg/L
4. CRP 10-15 mg/L
5. **Troponin**

To standardise the many troponin assays, we will scale the results using the ratio of the observed troponin value divided by the ULN for that particular troponin assay. For example, a patient with a troponin value of 96 using an assay which has an ULN of 40 would have a scaled result of 96/40 = 2.4 xULN.

In clinical practice, troponin levels are frequently dichotomised into “positive” (meaning above ULN) or “negative”. We will dichotomise the peak troponin level as being either positive or negative based on the ULN for each troponin assay.

All analyses on troponin will be performed using the peak troponin level. For patients who have a single troponin measurement, the peak troponin will be based on this measurement. In the remainder of the patients who have more than one troponin test in the same hospital episode, the peak troponin value will be defined as the highest of all measurements.

#### Acute coronary syndrome diagnosis

The ICD-10 codes in Table 2 will be used to indicate an acute coronary syndrome (ACS) diagnosis.

| **ICD-10 Code** | **Category** |
| --- | --- |
| I20.0 | Unstable angina |
| I21.0 | Acute transmural myocardial infarction of anterior wall |
| I21.1 | Acute transmural myocardial infarction of inferior wall |
| I21.2 | Acute transmural myocardial infarction of other sites |
| I21.3 | Acute transmural myocardial infarction of unspecified site |
| I21.4 | Acute subendocardial myocardial infarction |
| I21.9 | Acute myocardial infarction, unspecified |
| I22.0 | Subsequent myocardial infarction of anterior wall |
| I22.1 | Subsequent myocardial infarction of inferior wall |
| I22.8 | Subsequent myocardial infarction of other sites |
| I22.9 | Subsequent myocardial infarction of unspecified site |
| I24.8 | Other forms of acute ischaemic heart disease |
| I24.9 | Acute ischaemic heart disease, unspecified |

**Table 2.** ICD-10 codes used to indicate an ACS diagnosis

#### Follow-up

- Using a retrospective cohort study design, all patients will be followed up until death or censoring on 1st April 2017.
- All-cause mortality will be the primary outcome. The nature of the data sources means that this is the outcome that will be available and it will be available with high fidelity.
- Life status will be ascertained using routinely collected data on the NHS Spine Application, which is linked to the Office of National Statistics, and thereby to the national registry of deaths.

## Statistical Methods

###

### Baseline data

Baseline and demographic characteristics of the four CRP groups will be summarised by standard descriptive summaries:

- means (standard deviation) for continuous variables which are normally distributed
- median (interquartile range) for continuous variables which are not normally distributed
- number (percentage) for categorical variables

Comparisons between CRP groups will be explored by Kruskal-Wallis one-way analysis of variance or χ2 test for trend.

The correlation between CRP and troponin concentrations will be assessed using the Pearson correlation coefficient.

**CRP level and mortality risk**

The Kaplan-Meier method will be used to calculate and display cumulative mortality, with CRP groups compared using the log-rank statistic. Kaplan-Meier mortality curves will be created for the following groups:

- 4 CRP groups
- 4 CRP groups stratified by troponin positive or negative
- 4 CRP groups stratified by troponin positive or negative in subgroups of troponin positive patients according to troponin level (troponin 1-5 xULN, 5-10 xULN, 10-100 xULN and >100 xULN)
- 4 CRP groups according to ACS diagnosis

Multivariate Cox regression analysis will be applied to investigate whether the CRP groups independently predict mortality after adjusting for demographic and clinical variables.

The proportional hazard assumption will be tested, with a violation indicated by a significant relationship between Schoenfield residuals of a covariate and time. If the proportional hazards assumption is violated, Cox regression analysis with time-dependent covariates will be used with follow-up time divided into time intervals within which the proportional hazard assumptions are met.

Furthermore, using Martingale residuals, if non-linearity is detected in the relationship between the log hazard and a continuous covariate, the non-linear relationship will be modelled using restricted cubic splines.

**CRP risk model discrimination and reclassification**

To assess the predictive role of CRP on short- and long-term mortality beyond conventional risk factors and troponin, three statistical models will be established:

- model 1: age, gender, haemoglobin and creatinine
- model 2: model 1 + troponin (negative or positive)
- model 3: model 2 + CRP (CRP <2 mg/L, 2-4.9 mg/L, 5-9.9 mg/L and 10-15 mg/L)

Discrimination of the different models will be assessed by comparing the areas under the receiver operating characteristic curves (AUROC) using the nonparametric approach of DeLong et al [1].

The continuous net reclassification index (NRI) and integrated discrimination improvement (IDI) analysis will be performed with the censored survival data [2].

**Relationship between negative predictive value of CRP and troponin testing with hypothetical mortality**

The negative predictive values of a negative CRP, negative troponin or both a negative troponin and CRP will be plotted against the range of hypothetical mortality rates at short- and long-term follow-up.

### Statistical significance

All hypothesis tests will be 2-tailed. A p-value of <0.05 will be considered statistically significant. No correction will be implemented for multiple testing.

### Statistical package

Statistical analyses will be performed using SPSS software version 24 (SPSS Inc., Chicago, Illinois, United States), R 3.3.2 statistical package (the R Core Team, Vienna, Austria) or MedCalc version 15.8 (MedCalc Software, Mariakerke, Belgium).

**References**

[1] DeLong ER, DeLong DM, Clarke-Pearson DL. Comparing the areas under two or more correlated receiver operating characteristic curves: a nonparametric approach. *Biometrics* 1988;44:837-45.

[2] survIDINRI: IDI and NRI for Comparing Competing Risk Prediction Models with Censored Survival Data; Version R package version 1.1-1. Available online: https://cran.r-project.org/web/packages/survIDINRI/survIDINRI.pdf (accessed on 25 December 2021).
