# Supplementary material for: Mortality risk prediction of high-sensitivity C-reactive protein in suspected acute coronary syndrome: A cohort study
Source: PLoS Med. 2022 Feb 22;19(2):e1003911. doi: 10.1371/journal.pmed.1003911 (PMC8863282; doi:10.1371/journal.pmed.1003911)
Supplement: S1 Table — (DOCX) [file pmed.1003911.s004.docx]

**S1 Table. Troponin assays at participating cardiac centres**

| **S1 Table.** Troponin assays at participating cardiac centres | | | |  |
| --- | --- | --- | --- | --- |
| **Assay Manufacturer / Platform** | **Gender-specific** | **99^th^ percentile of the ULN (ng/L)** | **Troponin** | **Number of patients** |
| Roche Elecsys cTnT | No | 10 | Tn T | 347 |
| Roche Cobas | No | 14  (results reported with ULN of 10) | Tn T | 17671 |
| Roche Elecsys | No | 14  (results reported with ULN of 13) | Tn T | 43112 |
| Abbott i-STAT | Female | 15 | Tn I | 10981 |
| Siemens Centaur XP | No | 16 | Tn I | 22354 |
| Roche Elecsys cTnT | No | 30 | Tn T | 9420 |
| Abbott cTnI | No | 32 | Tn I | 50718 |
| Abbott i-STAT | Male | 34 | Tn I | 12853 |
| Abbott Architect | No | 40 | Tn I | 78037 |
| Roche Cardiac Reader | No | 50 | Tn T | 947 |
| **Number of patients refer to all 257948 patients who had a troponin measured** | | | |  |
