# Supplementary material for: Mortality risk prediction of high-sensitivity C-reactive protein in suspected acute coronary syndrome: A cohort study
Source: PLoS Med. 2022 Feb 22;19(2):e1003911. doi: 10.1371/journal.pmed.1003911 (PMC8863282; doi:10.1371/journal.pmed.1003911)
Supplement: S2 Table — ACS, acute coronary syndrome; ICD, International Classification of Diseases. (DOCX) [file pmed.1003911.s005.docx]

**S2 Table. ICD-10 diagnostic codes used to indicate an acute coronary syndrome diagnosis**

| S2 Table. ICD-10 diagnostic codes used to indicate an acute coronary syndrome diagnosis | | | | |
| --- | --- | --- | --- | --- |
| ICD-10 Code | **Category** |  |  |  |
| I20.0 | Unstable angina | | | |
| I21.0 | Acute transmural myocardial infarction of anterior wall | | | |
| I21.1 | Acute transmural myocardial infarction of inferior wall | | | |
| I21.2 | Acute transmural myocardial infarction of other sites | | | |
| I21.3 | Acute transmural myocardial infarction of unspecified site | | | |
| I21.4 | Acute subendocardial myocardial infarction | | | |
| I21.9 | Acute myocardial infarction, unspecified | | | |
| I22.0 | Subsequent myocardial infarction of anterior wall | | | |
| I22.1 | Subsequent myocardial infarction of inferior wall | | | |
| I22.8 | Subsequent myocardial infarction of other sites | | | |
| I22.9 | Subsequent myocardial infarction of unspecified site | | | |
| I24.8 | Other forms of acute ischaemic heart disease | | | |
| I24.9 | Acute ischaemic heart disease, unspecified | | | |
| ICD, International Statistical Classification of Diseases and Related Health Problems. | | | | |
