# Supplementary material for: Mortality risk prediction of high-sensitivity C-reactive protein in suspected acute coronary syndrome: A cohort study
Source: PLoS Med. 2022 Feb 22;19(2):e1003911. doi: 10.1371/journal.pmed.1003911 (PMC8863282; doi:10.1371/journal.pmed.1003911)
Supplement: S3 Table — hsCRP, high-sensitivity C-reactive protein. (DOCX) [file pmed.1003911.s006.docx]

**S3 Table. hsCRP assays at participating cardiac centres**

| S3 Table. hsCRP assays at participating cardiac centres | | | | |
| --- | --- | --- | --- | --- |
| Assay Manufacturer - Platform | **99^th^ percentile of the ULN (mg/L)** | **Limit of detection (mg/L)** | **Number of patients (Cardiac Centre)** | **Assay Manufacturer - Platform** |
| Roche - Cobas | 4 | 0.1  (reported <1 mg/L) | 19076 (GSTT) | Roche - Cobas |
| Abbott - Architect | 5 | 0.1 | 24295 (ICHT)  33623 (OUH)  14524 (UCL) | Abbott - Architect |
| Abbott - Architect | 10 | 0.2 | 10819 (ICHT) | Abbott - Architect |
| GSTT; Guy’s and St Thomas’ NHS Foundation Trust; ICHT, Imperial College Healthcare NHS Trust; OUH, Oxford University Hospitals NHS Foundation Trust; UCL, University College London Hospitals NHS Foundation Trust; ULN, 99^th^ percentile of the upper limit of normal. | | | | |
