# Supplementary material for: Mortality risk prediction of high-sensitivity C-reactive protein in suspected acute coronary syndrome: A cohort study
Source: PLoS Med. 2022 Feb 22;19(2):e1003911. doi: 10.1371/journal.pmed.1003911 (PMC8863282; doi:10.1371/journal.pmed.1003911)
Supplement: S4 Table — HR, hazard ratio; hsCRP, high-sensitivity C-reactive protein. (DOCX) [file pmed.1003911.s007.docx]

**S4 Table. Adjusted hazard ratios for 3-year mortality according to troponin and hsCRP stratified groups**

| **S4 Table.** Adjusted hazard ratios for 3-year mortality according to troponin and hsCRP stratified groups | | | | |
| --- | --- | --- | --- | --- |
| **hsCRP (mg/dL)** | **Troponin level** | **Number of patients** | **Hazard ration (95% confidence interval)** | **P-value** |
| <2 | Negative | 32291 | Reference | - |
| 2–4.9 | Negative | 21460 | 1.48 (1.38 – 1.58) | <0.001 |
| 5–9.9 | Negative | 19555 | 1.72 (1.60 – 1.84) | <0.001 |
| 10–15 | Negative | 6467 | 2.51 (2.32 – 2.72) | <0.001 |
|  |  |  |  |  |
| <2 | Positive | 6099 | 1.75 (1.61 – 1.90) | <0.001 |
| 2–4.9 | Positive | 5937 | 2.18 (2.02 – 2.36) | <0.001 |
| 5–9.9 | Positive | 7402 | 2.45 (2.28 – 2.63) | <0.001 |
| 10–15 | Positive | 3126 | 3.47 (3.20 – 3.77) | <0.001 |
|  |  |  |  |  |
| **Multivariable Cox regression analysis was adjusted for age, gender, haemoglobin, white cell count, platelet count, creatinine. The proportional hazards assumption was met over 3 years across the 8 groups.** | | | | |
