# Supplementary material for: Mortality risk prediction of high-sensitivity C-reactive protein in suspected acute coronary syndrome: A cohort study
Source: PLoS Med. 2022 Feb 22;19(2):e1003911. doi: 10.1371/journal.pmed.1003911 (PMC8863282; doi:10.1371/journal.pmed.1003911)
Supplement: S1 Fig — hsCRP, high-sensitivity C-reactive protein. (DOCX) [file pmed.1003911.s008.docx]

**S1 Figure. Correlation between hsCRP and troponin level**

| S1 Figure. Correlation between hsCRP and troponin level |
| --- |
|  |
| Pearson correlation R=0.019, p<0.0001. hsCRP, high-sensitivity C-reactive protein; ULN, 99^th^ percentile of the upper limit of normal. |
