# Supplementary material for: Mortality risk prediction of high-sensitivity C-reactive protein in suspected acute coronary syndrome: A cohort study
Source: PLoS Med. 2022 Feb 22;19(2):e1003911. doi: 10.1371/journal.pmed.1003911 (PMC8863282; doi:10.1371/journal.pmed.1003911)
Supplement: S2 Fig — hsCRP, high-sensitivity C-reactive protein. (DOCX) [file pmed.1003911.s009.docx]

**S2 Figure. Kaplan-Meier mortality curve by hsCRP level and different levels of troponin positivity**

| **S2 Figure.** Kaplan-Meier mortality curve by hsCRP level and different levels of troponin positivity | |
| --- | --- |
| **A Troponin 1-5 xULN**   | **B Troponin 5-10 xULN**   |
| **C Troponin 10-100 xULN**   | **D Troponin >100 xULN**   |
| **hsCRP, high-sensitivity C-reactive protein; Tn +, positive troponin; Tn –, negative troponin; ULN, 99^th^ percentile of the upper limit of normal.** | |
