# Supplementary material for: Mortality risk prediction of high-sensitivity C-reactive protein in suspected acute coronary syndrome: A cohort study
Source: PLoS Med. 2022 Feb 22;19(2):e1003911. doi: 10.1371/journal.pmed.1003911 (PMC8863282; doi:10.1371/journal.pmed.1003911)
Supplement: S3 Fig — ACS, acute coronary syndrome. (DOCX) [file pmed.1003911.s010.docx]

**S3 Figure. Unadjusted Kaplan-Meier mortality curves by ACS diagnosis**

| **S3 Figure.** Unadjusted Kaplan-Meier mortality curves by ACS diagnosis |
| --- |
| **** |
| **ACS, acute coronary syndrome; hsCRP, high-sensitivity C-reactive protein.** |
