# Supplementary material for: Mortality risk prediction of high-sensitivity C-reactive protein in suspected acute coronary syndrome: A cohort study
Source: PLoS Med. 2022 Feb 22;19(2):e1003911. doi: 10.1371/journal.pmed.1003911 (PMC8863282; doi:10.1371/journal.pmed.1003911)
Supplement: Attachment — Submitted filename: Response to Reveiwer Document.docx [file pmed.1003911.s011.docx]

1) In revising the manuscript for further consideration, your revisions should address the specific points made by each reviewer and the editors. Please also check the guidelines for revised papers at http://journals.plos.org/plosmedicine/s/revising-your-manuscript for any that apply to your paper. In your rebuttal letter you should indicate your response to the reviewers' and editors' comments, the changes you have made in the manuscript, and include either an excerpt of the revised text or the location (eg: page and line number) where each change can be found. Please submit a clean version of the paper as the main article file; a version with changes marked should be uploaded as a marked up manuscript.

Many thanks for inviting us to revise our manuscript entitled “High sensitivity C-reactive protein predicts mortality beyond troponin in 102337 patients with suspected acute coronary syndrome”. We have uploaded two versions of the revised manuscript, with and without tracked changes.

2) In addition, we request that you upload any figures associated with your paper as individual TIF or EPS files with 300dpi resolution at resubmission; please read our figure guidelines for more information on our requirements: http://journals.plos.org/plosmedicine/s/figures. While revising your submission, please upload your figure files to the PACE digital diagnostic tool, https://pacev2.apexcovantage.com/. PACE helps ensure that figures meet PLOS requirements. To use PACE, you must first register as a user. Then, login and navigate to the UPLOAD tab, where you will find detailed instructions on how to use the tool. If you encounter any issues or have any questions when using PACE, please email us at [PLOSMedicine@plos.org](mailto:PLOSMedicine@plos.org).

We can confirm that all figures in the manuscript document have been adjusted to satisfy the PACE image requirements.

3) We ask every co-author listed on the manuscript to fill in a contributing author statement, making sure to declare all competing interests. If any of the co-authors have not filled in the statement, we will remind them to do so when the paper is revised. If all statements are not completed in a timely fashion this could hold up the re-review process. If new competing interests are declared later in the revision process, this may also hold up the submission. Should there be a problem getting one of your co-authors to fill in a statement we will be in contact.  YOU MUST NOT ADD OR REMOVE AUTHORS UNLESS YOU HAVE ALERTED THE EDITOR HANDLING THE MANUSCRIPT TO THE CHANGE AND THEY SPECIFICALLY HAVE AGREED TO IT. You can see our competing interests policy here: <http://journals.plos.org/plosmedicine/s/competing-interests>.

All authors have previously completed the contributing author statement with confirmation of no competing interests.

4) To enhance the reproducibility of your results, we recommend that you deposit your laboratory protocols in protocols.io, where a protocol can be assigned its own identifier (DOI) such that it can be cited independently in the future. Additionally, PLOS ONE offers an option to publish peer-reviewed clinical study protocols. Read more information on sharing protocols at https://plos.org/protocols?utm_medium=editorial-email&utm_source=authorletters&utm_campaign=protocols

We have uploaded our data collection, data access and data analysis plan as an online supplement accessible via the ‘supporting information’ section.

5) Please ensure that the paper adheres to the PLOS Data Availability Policy (see http://journals.plos.org/plosmedicine/s/data-availability), which requires that all data underlying the study's findings be provided in a repository or as Supporting Information. For data residing with a third party, authors are required to provide instructions with contact information for obtaining the data. PLOS journals do not allow statements supported by "data not shown" or "unpublished results." For such statements, authors must provide supporting data or cite public sources that include it.

We have updated the data availability policy to state that “the datasets generated or analysed, or both, during this study are not publicly available because of governance restrictions.”

**Requests from the editors**

1) Please revise your title according to PLOS Medicine's style. Your title must be nondeclarative and not a question. It should begin with main concept if possible. "Effect of" should be used only if causality can be inferred, i.e., for an RCT. Please place the study design ("A randomized controlled trial," "A retrospective study," "A modelling study," etc.) in the subtitle (ie, after a colon).

The title has been amended in accordance with PLOS Medicine’s author guidance.

**Title**

*The mortality risk prediction of mildly elevated high-sensitivity C-reactive protein beyond troponin in 102337 patients with suspected acute coronary syndrome (NIHR Health Informatics Collaborative CRP-RISK Study): A retrospective cohort study*

2) Please structure your abstract using the PLOS Medicine headings (Background, Methods and Findings, Conclusions).

   Abstract Background:
   Provide the context of why the study is important. The final sentence should clearly state the study question.

   Abstract Methods and Findings:
   * Please include the important dependent variables that are adjusted for in the analyses.
   * In the last sentence of the Abstract Methods and Findings section, please describe the main limitation(s) of the study's methodology.

Apologies for not structuring the abstract in accordance with PLOS Medicine headings. We have now amended the abstract using the correct abstract structure.

Abstract

**Background**

There is limited evidence on the use of high-sensitivity CRP (hsCRP) as a biomarker for selecting patients for advanced cardiovascular therapies in the modern era. The prognostic value of mildly elevated hsCRP beyond troponin in a large real-world cohort of unselected patients presenting with suspected acute coronary syndromes (ACS) is unknown. We evaluated whether a mildly elevated hsCRP (up to 15 mg/L) was associated with mortality risk, beyond troponin level, in patients with suspected ACS.

**Methods and Findings**

We conducted a retrospective cohort study based on National Institute for Health Research Health Informatics Collaborative data of 257,948 patients with suspected ACS who had a troponin measured at five cardiac centres in the United Kingdom between 2010 and 2017. We excluded patients with clinically abnormal white cell counts (WCCs) and hsCRP >15 mg/L to limit the population to those without overt infections, malignancies or systemic inflammatory conditions. Patients were divided into four hsCRP groups (<2, 2-4.9, 5-9.9 and 10-15 mg/L). Troponin levels were standardised as a multiple of each laboratory’s 99^th^ percentile of the upper limit of normal. Patients were classified as ACS based on assigned discharge codes. The main outcome measure was mortality within 3 years of index presentation.

The association between hsCRP levels and all-cause mortality was assessed using multivariable Cox regression analysis adjusted for age, gender, haemoglobin, white cell count, platelet count, creatinine and troponin. All analyses on hsCRP and other haematological and biochemical blood tests, apart from troponin, were performed using the first result measured during the hospital care episode. All analyses on troponin were performed using the peak troponin level.

Following the exclusion criteria, there were 102,337 patients included in the analysis (hsCRP <2 mg/L (n=38,390), 2-4.9 mg/L (n=27,397), 5-9.9 mg/L (n=26,957) and 10-15 mg/L (n=9,593)). On multivariable Cox regression analysis, there was a positive and graded relationship between hsCRP level and mortality at baseline, which remained at three-years (hazard ratio (95% CI) of 1.32 (1.18-1.48) for those with hsCRP 2.0-4.9mg/L, and 1.40 (1.26-1.57), and 2.00 (1.75-2.28) for those with hsCRP 5-9.9 mg/L and 10-15 mg/L, respectively. This relationship was independent of troponin in all suspected ACS patients, and was further verified in those who were confirmed to have an ACS diagnosis by clinical coding.

The study had access to all-cause, but not specific, mortality data. This limits our ability to understand the cause of death in the patients with higher hsCRP levels. However, the exclusion of those with abnormal WCC or hsCRP levels >15mg/L makes it unlikely that sepsis was a major contributor to the excess mortality.

**Conclusion**

These multi-centre, real-world data from a large cohort of patients with suspected ACS identify mildly elevated hsCRP (up to 15 mg/L) as a clinically meaningful prognostic marker beyond troponin and point to its potential utility in selecting patients for novel treatments targeting inflammation.

3) Financial disclosure: your financial disclosure does not detail the funding source, please provide this information in your response to the submission form. The detailed funding information at the end of the main text should be removed and provided instead as part of your response to the submission form (this will then be published as metadata in the event of publication).

The submission form has been updated with funding information.

4) Similar to the above, please remove the ‘Authors’ Contributions’ and ‘Conflict of Interest’ sections from the end of the main text and ensure this information is captured in your submission form (this is essential as your submission form currently states no competing interests exist which is at odds with the main text).

The submission form has been updated with conflict of interest information. Unfortunately, we were unable to located the ‘authors’ contribution’ section on the submission form. We have left this section at the end of the main text.

5) You state in your Data Availability Statement that some restrictions will apply but also then state that all data is contained within the manuscript and/or supporting information files – please update for consistency, bearing in mind our data availability requirements (<https://journals.plos.org/plosmedicine/s/data-availability)>.

Apologies for this confusion. We have updated the data availability statement with the following:

*The datasets generated or analysed, or both, during this study are not publicly available because of governance restrictions.*

6) At this stage, we ask that you include a short, non-technical Author Summary of your research to make findings accessible to a wide audience that includes both scientists and non-scientists. The Author Summary should immediately follow the Abstract in your revised manuscript. This text is subject to editorial change and should be distinct from the scientific abstract. Please see our author guidelines for more information: <https://journals.plos.org/plosmedicine/s/revising-your-manuscript#loc-author-summary>

***Author summary***

***Why was this study done?***

*C-reactive protein (CRP) is a substance produced by the liver in response to inflammation. In patients presenting with a suspected heart attack, low levels of CRP on admission may prove useful in predicting death beyond using troponin, a marker of heart muscle damage.*

***What did the researchers do and find?***

*In this study of 102,337 patients with suspected heart attack, the higher the CRP level, the higher the risk of death. This relationship was independent of the troponin level in all patients with suspected heart attack, and was further verified in those who were confirmed to have a heart attack diagnosis by clinical coding.*

***What do these findings mean?***

*CRP is a clinically meaningful marker of risk of death in addition to troponin in patients with suspected heart attack and point to its potential utility in selecting patients for new treatments which target reducing inflammation.*

7) Please update your manuscript to include line numbering in the margin.

Line numbering has been added to the margin of the manuscript.

8) Please update your citations to be in square brackets.

We have updated the citations in the manuscript to be in square brackets.

9) Please relocate the information pertaining to the ethical approval from the end of the main text to the methods section.

The ‘ethical approval’ section has been relocated to the methods section.

10) I could not locate your data acquisition and statistical analysis plan? Please ensure these are included and, when stating that the data acquisition and statistical analysis plan are available in the supplementary material, please reference the relevant section of the supplementary material (e.g. S1).
11) Please only report P values to 3 decimal places (P<0.001 rather than P<0.0001).

We have updated all P-values to 3 decimal places throughout the manuscript.

12) Your study is observational and therefore causality cannot be inferred. Please remove language that implies causality, such as ‘confirms its value as a biomarker’. Refer to associations instead or tone down the language used (e.g. suggests, potential, etc.).

Thank you for this suggestion. We have reworded the language throughout the manuscript to avoid implication of causality with our results.

13) Please add ‘to our knowledge’ when stating that ‘there have been no studies that have demonstrated significantly superior AUROC’ in paragraph 5 of your discussion.

Many thanks for this suggestion. The sentence has been updated to your recommendations.

14) Please remove the One-sentence summary.

The one-sentence summary has been removed, as requested.

15) References: journal name abbreviations should be those found in the National Center for Biotechnology Information (NCBI) databases.

The references have been revised in accordance with PLOS Medicine guidelines.

16) Please ensure that the study is reported according to the STROBE guideline, and include the completed STROBE checklist as Supporting Information. Please add the following statement, or similar, to the Methods: "This study is reported as per the Strengthening the Reporting of Observational Studies in Epidemiology (STROBE) guideline (S1 Checklist)."

    The STROBE guideline can be found here: http://www.equator-network.org/reporting-guidelines/strobe/

    When completing the checklist, please use section and paragraph numbers, rather than page numbers.

We have attached the completed STROBE checklist as Supporting Information.

**Comments from the reviewers:**

Reviewer #1

"High sensitivity C-reactive protein predicts mortality beyond troponin in 102337 patients with suspected acute coronary syndrome" studies the association of (mildly) elevated levels of high-sensitivity C-reactive protein (hsCRP) on long-term (3 year) mortality risk in patients with suspected acute coronary syndromes (ACS), independent of troponin level. The main analysis concluded that there was indeed a positive and graded relationship between hsCRP and mortality at baseline and over three years, as summarized in Figures 2B and 3. In particular, stratification by hsCRP level demonstrated increasing 3-year mortality risk given fixed troponin positivity/negativity status (Figure 2B), and increased mortality over the full 3-year period by multivariable time-dependent Cox regression mortality analysis (Figure 3). hsCRP was therefore claimed to be a possible clinically prognostic marker in addition to troponin.

While the primary association between hsCRP and mortality risk appears clear, a number of issues might be considered, largely towards clarifying the methodology:

1. The definition of the upper limit of normal (ULN) for troponin assays might be explicitly stated, and the significance of "99th percentile of ULN" explained, especially since levels of troponin >100x ULN appear possible from Supplementary Figure 2. In particular, if ULN is the threshold on troponin level that separates troponin positive and negative diagnoses, then it would appear more natural to refer to the "99th percentile of troponin values" (to eliminate outliers at the upper end?); this might not be obvious to the more general reader.

Moreover, it is stated that the different troponin assays from various academic centres were standardized by scaling results using the ratio of observed troponin value divided by the (unique) ULN for each troponin assay. It might be clarified as to how the ULN for each troponin assay was determined. Is this ULN inherent to the troponin assay model, or empirically calculated on some cohort? Also, were such (scaling) normalizations significant?

The study dataset comprised patients who had their troponin level measured at one of five academic centres. We standardised the different troponin assays between the academic centres by scaling the results using the ratio of the observed troponin value divided by the 99^th^ percentile of the upper limit of normal (ULN) for each troponin assay. Each centre measured troponin I or troponin T using either contemporary or high sensitivity assays. These tests yielded results in different measurable ranges, with unique cut-off points for the ULN.

There is current lack of standardization of the various cardiac troponin assays as a physiological threshold for myocardial injury [23,24]. The ULN provided by the manufacturer for each troponin assay provides an analytical cut-off between normal and increased cardiac troponin levels.

We have updated the methods section with this additional level of detail and provided a Table in the supplementary material which displays the ULN for the troponin assays used at the participating cardiac centres.

**Page 9, line 4**

**Methods**

We standardised the different troponin assays between the academic centres by scaling the results using the ratio of the observed troponin value divided by the ULN for each troponin assay. Each centre measured troponin I or troponin T using either contemporary or high sensitivity assays (Supplementary Table 1). These tests yielded results in different measurable ranges, with unique cut-off points for the ULN. There is current lack of standardization of the various cardiac troponin assays as a physiological threshold for myocardial injury [23, 24]. The ULN provided by the manufacturer for each troponin assay provides an analytical cut-off between normal and increased cardiac troponin levels.  A positive troponin was defined as a result above the ULN for each troponin assay.

**References**

23.	Wildi K, Gimenez MR, Twerenbold R, Reichlin T, Jaeger C, Heinzelmann A, et al. Misdiagnosis of Myocardial Infarction Related to Limitations of the Current Regulatory Approach to Define Clinical Decision Values for Cardiac Troponin. Circulation. 2015;131(23):2032-40. Epub 2015/05/08. doi: 10.1161/circulationaha.114.014129. PubMed PMID: 25948541; PubMed Central PMCID: PMCPMC4456170.

24.	Apple FS, Collinson PO. Analytical characteristics of high-sensitivity cardiac troponin assays. Clinical chemistry. 2012;58(1):54-61. Epub 2011/10/04. doi: 10.1373/clinchem.2011.165795. PubMed PMID: 21965555.

**Supplementary material**

| **Supplementary Table 1.** Troponin assays at participating cardiac centres | | | |  |
| --- | --- | --- | --- | --- |
| **Assay Manufacturer / Platform** | **Gender-specific** | **99^th^ percentile of the ULN (ng/L)** | **Troponin** | **Number of patients** |
| Roche Elecsys cTnT | No | 10 | Tn T | 347 |
| Roche Cobas | No | 14  (results reported with ULN of 10) | Tn T | 17671 |
| Roche Elecsys | No | 14  (results reported with ULN of 13) | Tn T | 43112 |
| Abbott i-STAT | Female | 15 | Tn I | 10981 |
| Siemens Centaur XP | No | 16 | Tn I | 22354 |
| Roche Elecsys cTnT | No | 30 | Tn T | 9420 |
| Abbott cTnI | No | 32 | Tn I | 50718 |
| Abbott i-STAT | Male | 34 | Tn I | 12853 |
| Abbott Architect | No | 40 | Tn I | 78037 |
| Roche Cardiac Reader | No | 50 | Tn T | 947 |
| **Number of patients refer to all 257948 patients who had a troponin measured** | | | |  |

2. Supplementary Figure 2 shows sensitivity analyses comparing the mortality curves for different definitions of troponin positivity (1-5x ULN, 5-10x, 10-100x, >100x). It appears that overall mortality risk is generally highest for 5-10x ULN, and actually lower for >100x ULN, than 1-5x ULN, i.e. the association of troponin level with mortality risk is nonlinear (as also briefly raised as an interesting point in the Discussion section). If so, it might be discussed in greater detail as to whether this nonlinear association is expected, and its possible causes.

Thank you for highlighting this interesting observation. While there was a graded relationship between hsCRP level and mortality amongst patients with different levels of troponin positivity, the mortality risk in the hsCRP groups increased progressively up to >5-10×ULN of troponin and then progressively declined in the higher troponin groups. Previous research from our group has demonstrated this inverted U shaped relation between troponin level and mortality in an unselected cohort of patients undergoing troponin testing. This was primarily due to an interaction between ACS diagnosis and differential receipt of invasive management in this cohort. We have described this finding in our discussion, as well as provided a reference to our previous paper where the unexpected inverted U shaped relationship between troponin level and mortality is discussed in detail.

**Page 18, line 4**

***Discussion***

*Moreover, an interesting finding in the present study is that those with raised hsCRP and moderate troponin elevations (Supplementary Figure 2) had the greatest mortality risk. While there was a graded relationship between hsCRP level and mortality amongst patients with different levels of troponin positivity, the mortality risk in the hsCRP groups increased progressively up to >5-10×ULN of troponin and then progressively declined in the higher troponin groups. Previous research from our group has demonstrated this inverted U-shaped relation between troponin level and mortality in an unselected cohort of patients undergoing troponin testing. This was primarily due to an interaction between ACS diagnosis and differential receipt of invasive management in this cohort. [20] The >5-10×ULN troponin group are likely to represent non-ST segment myocardial infarctions (NSTEMI) than STEMI, and it is perhaps in this population that any targeted anti-inflammatory therapy would have the most clinical benefit.*

***References***

*20.	Kaura A, Arnold AD, Panoulas V, Glampson B, Davies J, Mulla A, et al. Prognostic significance of troponin level in 3121 patients presenting with atrial fibrillation (The NIHR Health Informatics Collaborative TROP-AF study). Journal of the American Heart Association. 2020;9(7):e013684. Epub 2020/03/28. doi: 10.1161/jaha.119.013684. PubMed PMID: 32212911.*

3. It is stated that the data model involves patients with suspected ACS, as characterized by the request of a troponin test. It might be clarified as to whether this set of patients with troponin test results corresponds to the n=257,948 patients of the initial cohort (Figure 1), and whether the troponin test is exclusively administered towards suspected ACS.

Apologies for any confusion regarding the study population definition. The study cohort was defined as those with suspected ACS characterised by the request of a troponin test. This initial cohort included 257,948 patients who had a troponin assessed across all five sites. The flow chart in Figure 1 has been updated with the full definition of the starting population.

***Figure 1: Flow of patients through the study***

*hsCRP, high-sensitivity C-reactive protein; WCC, white cell count. *suspected ACS characterised by the request of a troponin*

4. It is stated that n=134,517 patients were admitted to hospital and thus had ICD discharge codes, and patients were classified as ACS based on said ICD-10 codes. It might be clarified as to how and where this n=134,517 can be represented in the patient flowchart (Figure 1)

Thank you for this excellent suggestion. We have revised the flow chart to now display the percentage of patients in each hsCRP group who were admitted to hospital and the percentage who were subsequently diagnosed with an ACS during their inpatient stay. The results section has been updated in reference to these results.

**Page 12, line 12**

***Results***

***Baseline population characteristics***

*Overall, 53.3% (n=54,534) of patients in the study cohort were admitted to hospital. Amongst these patients, 10.8% (n=5,910) were coded with an ACS during their inpatient stay.*

***Figure 1: Flow of patients through the study***

***hsCRP, high-sensitivity C-reactive protein; WCC, white cell count. *suspected ACS characterised by the request of a troponin***

5. It is stated that three statistical risk models were developed for mortality risk prediction (Table 2). The details of these models might be clarified - i.e. are they linear/logistic regression models (and if so, their coefficients might be provided), or other machine learning models, etc. Also, the choice of variables to match for/adjust for (e.g. ethnicity not included) might be briefly justified.

The statistical models were based on the predicted probabilities of a logistic regression model. All key variables from Table 1 (age, gender, haemoglobin, creatinine, troponin and hsCRP) were used for the hsCRP risk model apart from ethnicity due to approximately 10% of patients having missing data. Furthermore, for those who had ethnicity recorded, approximately 15% had “other” recorded as an ethnic category, therefore making it less easy to interpret as part of a clinical risk model. We have updated the methods section with this additional level of detail.

**Page 11, line 2**

***Methods***

***Statistical analysis***

*The statistical models were based on the predicted probabilities of a logistic regression model. Ethnicity was not included in the risk model due to approximately 10% of patients having missing data. Furthermore, for those who had ethnicity recorded, approximately 15% had “other” recorded as an ethnic category, therefore making it less easy to interpret as part of a clinical risk model.*

6. For the Kaplan-Meier analysis in Figure 2, it might be explicitly stated if any matching/adjustment was attempted. The "No. at risk" matrices might also be briefly explained/defined.

While all Kaplan-Meier survival curves are unadjusted, we present the results of fully adjusted Cox regression survival analyses. We have now clarified in the title of Figure 2 that the Kaplan-Meier curves are “unadjusted”. We have also provided an explanation in the Figure legend of the definition of the term “number at risk”.

***Figure 2: Unadjusted Kaplan-Meier mortality curves by (A) hsCRP level, (B) hsCRP level and troponin positivity, and (C) hsCRP level in a subgroup of patients with ACS***

*The number at risk is the number of patients at risk immediately before the relevant time point, t. Being "at risk" means that the patient has not died before time t, and is not censored before or at time t.*

7. For Figure 3, it might be considered to provide additional time-dependent Cox regression mortality analysis charts stratified on troponin positivity/negativity, for consistency with the previous Kaplan-Meier analysis in Figure 2.

Thank you for this suggestion. We have provided an additional table summarising the results of the Cox regression analysis in the hsCRP groups stratified by troponin positivity. The proportional hazard assumption was met in each of these groups, therefore a model using time-varying coefficients was not necessary.

**Page 14, line 3**

***Results***

***Higher hsCRP predicts significantly increased mortality risk***

*The hazard ratios for mortality risk at 3-years in the hsCRP groups stratified by troponin positivity are shown in Supplementary Table 4.*

| ***Supplementary Table 4.*** *Adjusted hazard ratios for 3-year mortality according to troponin and hsCRP stratified groups* | | | | |
| --- | --- | --- | --- | --- |
| ***hsCRP (mg/dL)*** | ***Troponin level*** | ***Number of patients*** | ***Hazard ration (95% confidence interval)*** | ***P-value*** |
| *<2* | *Negative* | *32291* | *Reference* | *-* |
| *2–4.9* | *Negative* | *21460* | *1.48 (1.38 – 1.58)* | *<0.0001* |
| *5–9.9* | *Negative* | *19555* | *1.72 (1.60 – 1.84)* | *<0.0001* |
| *10–15* | *Negative* | *6467* | *2.51 (2.32 – 2.72)* | *<0.0001* |
|  |  |  |  |  |
| *<2* | *Positive* | *6099* | *1.75 (1.61 – 1.90)* | *<0.0001* |
| *2–4.9* | *Positive* | *5937* | *2.18 (2.02 – 2.36)* | *<0.0001* |
| *5–9.9* | *Positive* | *7402* | *2.45 (2.28 – 2.63)* | *<0.0001* |
| *10–15* | *Positive* | *3126* | *3.47 (3.20 – 3.77)* | *<0.0001* |
|  |  |  |  |  |
| ***Multivariable Cox regression analysis was adjusted for age, gender, haemoglobin, white cell count, platelet count, creatinine. The proportional hazards assumption was met over 3 years across the 8 groups.*** | | | | |

8. Further on Figure 3, the time-dependent analysis would appear to require data from patients at each follow-up interval over the full 3-year period, whereas there does not appear to be a description for the treatment of patients with missing/incomplete data, in Figure 1 and elsewhere. It might be clarified whether all required data was available for all patients involved, or if not, how missing data was handled (this extends to other covariates, other than hsCRP)

Thank you for bringing this to our attention. We have updated the terminology in the methods section to better explain the analysis approach. Multivariable Cox regression analysis was applied to investigate whether the ‘baseline’ hsCRP groups independently predict 3-year mortality after adjusting for other demographic and clinical variables. As the proportional hazard assumption was violated, follow-up time was divided into six intervals: <1 month, 1-3 months, 3-6 months, 6-12 months, 12-24 months and 24-36 months. The proportional hazard assumption was met in each of these time intervals. The coefficients calculated during each of these time intervals are based on the variables measured during the index hospital admission. The corrected terminology is to describe the analysis as Cox regression analysis with ‘time-varying coefficients’ rather than using time-dependent covariates.

**Page 10, line 16**

***Methods***

***Statistical analysis***

*Cox regression analysis with time-varying coefficients was therefore used with follow-up time divided into six intervals: <1 month, 1-3 months, 3-6 months, 6-12 months, 12-24 months and 24-36 months. The proportional hazard assumption was met in each of these time intervals. The coefficients calculated during each of these time intervals were based on the variables measured during the index hospital admission. Furthermore, using Martingale residuals, nonlinearity was detected in the relationship between the log hazard and all continuous covariates. To model these non-linear relationships, we used restricted cubic splines in the Cox proportional hazards model.*

***Figure 3: Multivariable Cox regression analysis with time-varying coefficients***

*hsCRP, high-sensitivity C-reactive protein. Hazard ratios are adjusted for age, gender, haemoglobin, WCC, platelet count, creatinine and troponin level. The adjusted hazard ratios were calculated during the following time periods: <1 month, 1-3 months, 3-6 months, 6-12 months, 12-24 months and 24-36 months. The hazard ratios are displayed at the end of each respective time period. The error bars depict 95% confidence intervals.*

9. Figure 4 might be explained in greater detail, with "negative predictive value" in particular being clarified.

Thank you for highlighting this important point. We have provided further detail in the methods section on the rationale for this test and the definition of the term ‘negative predictive value’.

**Page 11, line 19**

***Methods***

***Statistical analysis***

*In order to evaluate the performance of negative hsCRP and troponin values in predicting mortality, the negative predictive values of a negative hsCRP, negative troponin or both a negative troponin and hsCRP were plotted against the range of hypothetical mortality rates at 30-days and 3-years. The negative predictive value, which varies with the prevalence of disease (mortality) is defined as the probability of not dying when the test result is negative.*

10. In the Results section, it is stated that "...Although we tested hsCRP in relation to peak troponin available in predicting mortality, we established that the median number of days between first troponin test and hsCRP was 0 (IQR 0), indicating that our hsCRP and troponin values represent presentation values rather than the sequelae of the index event". The significance of this statement might be clarified further. Does it imply that peak troponin is generally attained on the first test?

The aim of this statement was to demonstrate that the admission hsCRP used represented the presentation value rather than the sequelae of the index event. As we used the peak troponin value for our analysis, we have updated the results to now calculate for patients with an elevated troponin result, the median number of days between the admission hsCRP and the peak troponin level. The result was 3 (interquartile range 0-5) days, which better supports our statement.

**Page 12, line 24**

***Results***

***Baseline population characteristics***

*Although we tested hsCRP in relation to ‘peak’ troponin available in predicting mortality, we established that for patients with an elevated troponin result, the median number of days between the first admission hsCRP measured and peak troponin level was 3 (IQR 0-5) days, suggesting that the admission hsCRP represents presentation values rather than the sequelae of the index event.*

11. The claim of "...The independence of hsCRP from all other available covariates including troponin in predicting mortality" in the Discussion section, might be supported with more specific data.

We agree that this statement is non-specific. We have elaborated our key findings at the start of the discussion using more specific data.

**Page 15, line 4**

***Discussion***

*The mortality risk associated with increased hsCRP was independent of age, sex, haemoglobin, WCC, platelet count, creatinine and troponin positivity, suggesting its value as a clinically useful biomarker in the context of suspected ACS. Compared to a normal hsCRP (<2 mg/L), a mildly raised hsCRP between 10-15 mg/L was associated with a 2.2-fold increased mortality risk at 30-days which persisted at 3 years.*

**Minor comments:**

12. The emphasis on "mildly elevated hsCRP" in describing association with mortality risk might be clarified; from Figure 2, it appears that the association exists over a large range of hsCRP elevation.

Apologies for not clarifying the term “mildly elevated hsCRP” when describing the association between mildly elevated hsCRP and mortality risk. We have carefully reviewed the abstract and manuscript in detail and clarified, where relevant, the term “mildly elevated hsCRP”.

**Page 3, line 4**

***Abstract***

***Background***

*The prognostic value of mildly elevated hsCRP beyond troponin in a large real-world cohort of unselected patients presenting with suspected acute coronary syndromes (ACS) is unknown. We evaluated whether a mildly elevated hsCRP (up to 15 mg/L) was associated with mortality risk, beyond troponin level, in patients with suspected ACS.*

**Page 4, line 16**

***Abstract
Conclusion***

*These multi-centre, real-world data from a large cohort of patients with suspected ACS identify mildly elevated hsCRP (up to 15 mg/L) as a clinically meaningful prognostic marker beyond troponin and point to its potential utility in selecting patients for novel treatments targeting inflammation.*

**Page 7, line 26**

***Introduction***

*In this study, we aim to evaluate the prognostic value of mildly elevated hsCRP (up to 15 mg/L) beyond troponin in a large real-world cohort of unselected patients presenting with suspected ACS. Our hypothesis was that a mildly elevated hsCRP would be associated with mortality risk, beyond troponin, in patients with suspected ACS.*

**Page 15, line 2**

***Discussion***

*In this large retrospective cohort study, we demonstrate a positive and graded relationship between mildly elevated hsCRP levels (up to 15 mg/L) and all-cause mortality over 3 years.*

**Page 15, line 8**

***Discussion***

*In this study, patients who had negative serum troponin but a hsCRP between 10-15 mg/L had equivalent mortality to those who were troponin-positive but had a normal hsCRP, <2 mg/dL, in Kaplan-Meier analysis at 3 years.*

13. Assessment of hsCRP independent of troponin level is also variously referred to as "beyond troponin" (in the title) and "above troponin" (in the abstract). It might be considered to standardize the terminology throughout, for clarity.

Apologies for these inconsistencies in our terminology. Throughout the manuscript, where relevant, we refer to the assessment of hsCRP “beyond troponin”.

***Title***

*The mortality risk prediction of mildly elevated high-sensitivity C-reactive protein beyond troponin in 102337 patients with suspected acute coronary syndrome (NIHR Health Informatics Collaborative CRP-RISK Study): A retrospective cohort study*

**Page 3, line 4**

***Abstract***

***Background***

*The prognostic value of mildly elevated hsCRP beyond troponin in a large real-world cohort of unselected patients presenting with suspected acute coronary syndromes (ACS) is unknown. We evaluated whether a mildly elevated hsCRP (up to 15 mg/L) was associated with mortality risk, beyond troponin level, in patients with suspected ACS.*

**Page 7, line 26**

***Introduction***

*In this study, we aim to evaluate the prognostic value of mildly elevated hsCRP (up to 15 mg/L) beyond troponin in a large real-world cohort of unselected patients presenting with suspected ACS. Our hypothesis was that a mildly elevated hsCRP would be associated with mortality risk, beyond troponin, in patients with suspected ACS.*

**Page 4, line 16**

***Conclusion***

*These multi-centre, real-world data from a large cohort of patients with suspected ACS identify mildly elevated hsCRP (up to 15 mg/L) as a clinically meaningful prognostic marker beyond troponin and point to its potential utility in selecting patients for novel treatments targeting inflammation.*

14. In the Statistical Analysis section, it is stated that "the IDI reflects the change in calculated risk for each individual"; it might be clarified as to whether this change is positive by default (i.e. improvement).

Thank you for raising this important point. The statistical analysis section of the methods has been updated with a more comprehensive description of the IDI as a reclassification measure.

**Page 11, line 13**

***Methods***

***Statistical analysis***

*The IDI reflects the change in discrimination slope (i.e., difference between the mean estimated risk for patients who died and those who survived) of the model with the new predictor compared to the model with only the established predictors. IDI (range −100 to 100 %) represents overall risk discrimination improvement.*

**Reviewer #2**

This paper describes the incremental long-term prognostic value of high-sensitivity C-reactive protein (hsCRP) above troponin in a large real-world cohort of unselected ACS patients. The objective was to determine if mildly elevated hsCRP was associated with mortality risk, irrespective of troponin level. Eligible patients from 2010 until 2017 were included retrospectively, utilizing NIH Research Health Informatics Collaborative data collected from five hospital centers in Great Britain. Patient selection was based on troponin measurements, normal white blood cell counts and a hsCRP up to 15 mg/L. Patients were followed up until death or censoring within 3 years. To standardize, the ratio of the observed peak troponin value was divided by the ULN for each troponin assay. 102337 patients were included in the analysis. The population was divided into four categories, those with a normal hsCRP <2mg/L, and three groups of incrementally increasing hsCRP (2-4.9 mg/L, 5-9.9 mg/L and 10-15 mg/L). On multivariable Cox regression analysis, there was a positive and graded relationship between hsCRP level and mortality at baseline, which remained at three-years, irrespective of troponin values.

1. The background for this mega-study is based on the hypothesis that inflammation is a contributory factor for the development of atherosclerosis, supported by RCTs, such as CANTOS, COLCOT and LoDoCo2, the former applying canakinumab and the two latter applying colchicine as anti-inflammatory agents, whereas no effect of methotrexate was obtained in CIRT. These trials were performed in patients with established coronary heart disease, with cardiovascular disease as a primary endpoint, reflecting progression of atherosclerotic disease, whereas the current study presents results related to total mortality, which also includes non-cardiac death, and, ideally, this should have been accounted for when discussing the significance of hsCRP as a marker of atherosclerotic disease.

As you correctly highlight, the CANTOS, COLCOT and LoDoCo2 trials assessed the effect of anti-inflammatory agents on modulating CRP in populations with established cardiovascular disease. Conversely, in our study, we assess the importance of low levels of hsCRP in predicting mortality in an unselected population with suspected ACS who have both known and unknown cardiovascular disease. We have added a paragraph to the introduction highlighting this point.

The present study had access to all-cause, but not specific, mortality data. In the limitations section, we have discussed how this limits our ability to understand the causes of death in the patients with the higher hsCRP levels. However, the exclusion of those with abnormal WCC or hsCRP levels >15mg/L makes it unlikely that sepsis was a major contributor to the excess mortality. Nonetheless, all-cause mortality is clearly a clinically relevant endpoint. This has been highlighted as a limitation in both the abstract and the limitations sections of the manuscript.

**Page 7, line 21**

***Introduction***

*However, in patients presenting with suspected ACS, levels of hsCRP may either reflect background atherosclerotic disease activity, or indeed also reflect the sequelae of the acute event. Nevertheless, hsCRP levels at presentation may prove useful in prognostication beyond routinely studied parameters, including troponin.*

**Page 4, line 10**

***Abstract***

***Methods and Findings***

*The study had access to all-cause, but not specific, mortality data. This limits our ability to understand the cause of death in the patients with higher hsCRP levels. However, the exclusion of those with abnormal WCC or hsCRP levels >15mg/L makes it unlikely that sepsis was a major contributor to the excess mortality.*

**Page 18, line 25**

***Discussion***

*There are some limitations to this study. The present study had access to all-cause, but not specific, mortality data. This limits our ability to understand the causes of death in the patients with the higher hsCRP levels. However, the exclusion of those with abnormal WCC or hsCRP levels >15mg/L makes it unlikely that sepsis was a major contributor to the excess mortality. Nonetheless, all-cause mortality is clearly a clinically relevant endpoint.*

2. As patients in the highest as compared to the lowest quartile of hsCRP are 10 years older, one would expect an increasing proportion of co-morbidity through the quartiles of this observational study. These limitations are essential, especially when relating the results to progressive atherosclerotic disease and recommending hsCRP for selection of high-risk patients that may benefit from anti-inflammatory treatment.

Thank you for highlighting this important issue. The median age demographic of patients progressively increases from the lowest to the highest quartile of hsCRP. We were unable to determine the patient comorbidities for our cohort. Whilst we were unable to adjust our analyses by patient comorbidities, all survival analyses were adjusted for age, which may also act as a surrogate marker of patient comorbidities, which are expected to increase in frequency with older age. We have added these points to the limitations section of the discussion.

**Page 19, line 4**

***Discussion***

*We were unable to determine the patient comorbidities for our cohort. The median age demographic of patients progressively increases from the lowest to the highest quartile of hsCRP. Whilst we were unable to adjust our analyses by patient comorbidities, all survival analyses were adjusted for age, which may also act as a surrogate marker of patient comorbidities, which are expected to increase in frequency with older age.*

3. Hs-CRP is an acute phase reactant and may not be a reliable indicator of risk, when not measured during steady state. However, the observation that mortality was greatest in patients with raised hs-CRP and moderate troponin elevations, might support the authors' claim. In short, discussion and limitations require more attention.

As mentioned by the reviewer, hsCRP is an acute phase reactant and may not be a reliable indicator of risk unless it’s measured during steady state. Although we tested hsCRP in relation to ‘peak’ troponin available in predicting mortality, we established that for patients with an elevated troponin result, the median number of days between the first admission hsCRP measured and peak troponin level was 3 (IQR 0-5) days, indicating that the admission hsCRP represents presentation values rather than the sequelae of the index event. The results section has been updated with this additional level of information.

Additionally, as eluded to by the reviewer, while there was a graded relationship between hsCRP level and mortality amongst patients with different levels of troponin positivity, the mortality risk in the hsCRP groups increased progressively up to >5-10×ULN of troponin and then progressively declined in the higher troponin groups. Previous research from our group has demonstrated this inverted U shaped relation between troponin level and mortality in an unselected cohort of patients undergoing troponin testing. This was primarily due to an interaction between ACS diagnosis and differential receipt of invasive management in this cohort. We have described this finding in our discussion, as well as provided a reference to our previous paper where the unexpected inverted U shaped relationship between troponin level and mortality is discussed in detail.

**Page 12, line 24**

***Results***

***Baseline population characteristics***

*Although we tested hsCRP in relation to ‘peak’ troponin available in predicting mortality, we established that for patients with an elevated troponin result, the median number of days between the first admission hsCRP measured and peak troponin level was 3 (IQR 0-5) days, suggesting that the admission hsCRP represents presentation values rather than the sequelae of the index event.*

**Page 18, line 4**

***Discussion***

*Moreover, an interesting finding in the present study is that those with raised hsCRP and moderate troponin elevations (Supplementary Figure 2) had the greatest mortality risk. While there was a graded relationship between hsCRP level and mortality amongst patients with different levels of troponin positivity, the mortality risk in the hsCRP groups increased progressively up to >5-10×ULN of troponin and then progressively declined in the higher troponin groups. Previous research from our group has demonstrated this inverted U-shaped relation between troponin level and mortality in an unselected cohort of patients undergoing troponin testing. This was primarily due to an interaction between ACS diagnosis and differential receipt of invasive management in this cohort. [21] The >5-10×ULN troponin group are likely to represent non-ST segment myocardial infarctions (NSTEMI) than STEMI, and it is perhaps in this population that any targeted anti-inflammatory therapy would have the most clinical benefit.*

***References***

*21.	Kaura A, Panoulas V, Glampson B, Davies J, Mulla A, Woods K, et al. Association of troponin level and age with mortality in 250 000 patients: cohort study across five UK acute care centres. BMJ (Clinical research ed). 2019;367:l6055. Epub 2019/11/22. doi: 10.1136/bmj.l6055. PubMed PMID: 31748235; PubMed Central PMCID: PMCPMC6865859*

**Reviewer #3**

This manuscript describes the importance of measuring hsCRP in ACS patients as prognostic factor in a large patient cohort. This research question is crucial to further provide information on patient selection for anti-inflammatory therapies and early prognosis of deleterious outcomes. 

The main claims are that CRP admission levels of suspected and actual ACS patients significantly correlate with 30-day and 3-year mortality of these patients. It is therefore concluded that CRP should be routinely assessed in ACS patients and not discarded as only a systemic inflammation marker. Together with troponin, risk assessment of ACS patients could be easily improved. 

Minor points:

1. The authors should discuss critically and in more detail that all data analysis is based on the initial hsCRP value at hospital admission. They write "that our hsCRP and troponin values represent presentation values rather than sequelae of the index event". The authors should put this in the context of recent publications regarding CRP's active role in the pathophysiology of acute coronary incidents such as AMI and the acute phase response in general. Is there information of the exact time point of CRP measurement and actual diagnosed ACS? This could enlighten whether it is actually the CRP baseline value or already the initial increase of the acute phase response.

It is difficult in clinical practice to identify the exact time point when an ACS event occurs. The time of diagnosis may not coincide with the time of onset of the ACS event. In order to determine the effect of baseline, steady state, hsCRP levels rather than hsCRP rises in response to an infarct, we carried out analyses on admission hsCRP levels in relation to ‘peak’ troponin available in predicting mortality. We established that for patients with an elevated troponin result, the median number of days between the first admission hsCRP measured and peak troponin level was 3 (IQR 0-5) days, indicating that the admission hsCRP represents presentation values rather than the sequelae of the index event. The results section has been updated with this additional level of information. We cite relevant references in response to your further points.

**Page 12, line 24**

***Results***

***Baseline population characteristics***

*Although we tested hsCRP in relation to ‘peak’ troponin available in predicting mortality, we established that for patients with an elevated troponin result, the median number of days between the first admission hsCRP measured and peak troponin level was 3 (IQR 0-5) days, suggesting that the admission hsCRP represents presentation values rather than the sequelae of the index event.*

**Page 16, line 22**

***Discussion***

*It is difficult in clinical practice to identify the exact time point when an ACS event occurs. The time of diagnosis may not coincide with the time of onset of the ACS event. In order to determine the effect of baseline hsCRP levels rather than hsCRP rises in response to an infarct, we carried out analyses on admission hsCRP levels in comparison to the ‘peak’ troponin level in predicting mortality. For patients with an elevated troponin result, the median number of days between the first admission hsCRP measured and peak troponin level was 3 (IQR 0-5) days, infering that the admission hsCRP levels likely represent presentation values rather than the sequelae of the index event.*

2. By excluding patients with CRP > 15 mg/L not only infections but also already ongoing acute coronary events were excluded. The authors also do not mention that in the CAMI-1 trial, a significant association was found between CRP gradient (CRP increase between 12 and 32 hours after symptom onset) and infarct size, LVEF, and circumferential and longitudinal strain. In addition, some publications reported a significant correlation between infarct size and peak CRP, which could also be used as a predictor (most from University of Innsbruck). Both could be discussed in relation to the use of CRP level on admission. In the context of a 2016 meta-analysis showing a significant association between infarct size and mortality (Stone et al.), another point could be discussed.

Thank you for your insight regarding how our results add to the current literature. We excluded individuals who had a CRP >15mg/L to limit the population to those without overt infections, malignancies or systemic inflammatory conditions. We don’t feel this has excluded many patients with on-going acute cardiac events. In a recent study investigating the association of initial hsCRP levels with major adverse cardiovascular events (MACE) and mortality after ACS (Mani P, et al, 2019), the median baseline hsCRP level was 10.5 mg/L (IQR, 4.2-30.3 mg/L) in ACS patients. On multivariable analysis, higher baseline hsCRP level (hazard ratio [HR], 1.36 [95% CI, 1.13-1.63]; *P* = 0.001) was also shown to be independently associated with MACE and mortality.

We have also now discussed our results in the context of the most recent literature highlighted by the reviewer. While we did not have access to data on serial CRP measurements, the correlation between the CRP gradient between 12 and 32 hours after symptom onset and both myocardial infarct size and the extent of reduction in left ventricular function in patients with ST elevation myocardial infarction in the CAMI-1 trial strongly indicates a pathogenic contribution of CRP in acute ACS. We further discuss whether CRP has a potential role as a mediator in addition to a marker of adverse cardiovascular events and mortality. The CAMI-1 investigators evaluated in this pilot study the feasibility and safety of CRP apheresis to reduce myocardial infarct size after STEMI as determined by cardiovascular magnetic resonance. While the primary endpoint of reduction of infarct size was not met, a sufficiently large randomized trial of CRP apheresis therapy in uncomplicated STEMI with the end point of infarct size reduction, is currently under development at the Medical University of Innsbruck. This should provide further insight as to whether CRP is also a potentially damaging mediator for cardiovascular disease, infarct size and subsequent mortality risk (Stone et al. 2016).

**Page 17, line 4**

***Discussion***

*We excluded individuals who had a CRP >15mg/L (or abnomral WCC) to limit the population to those without overt infections, malignancies or systemic inflammatory conditions. It is unlikely that we excluded many patients with ACS due to the low CRP levels measured in patients with ACS in previous studies. In a recent study investigating the association of initial hsCRP levels with major adverse cardiovascular events (MACE) and mortality after ACS [27], the median baseline hsCRP level was 10.5 mg/L (IQR, 4.2-30.3 mg/L) in ACS patients. On multivariable analysis, higher baseline hsCRP level (hazard ratio [HR], 1.36 [95% CI, 1.13-1.63]; P = 0.001) was also shown to be independently associated with MACE and mortality.*

*Recently, in the multi-centre matched-control pilot study on CRP apheresis in Acute Myocardial Infarction (CAMI-1), there was a correlation between the CRP gradient between 12 and 32 hours after symptom onset and both myocardial infarct size and the extent of reduction in left ventricular function [28]. The CAMI-1 investigators evaluated the feasibility and safety of CRP apheresis to reduce myocardial infarct size after STEMI as determined by cardiovascular magnetic resonance. While the primary endpoint of reduction of infarct size was not met, a sufficiently large randomized trial of CRP apheresis therapy in uncomplicated STEMI with the end point of infarct size reduction, is currently under development at the Medical University of Innsbruck. This should provide further insight as to whether CRP is also a potentially damaging mediator for cardiovascular disease, infarct size and subsequent mortality risk [29]. However, these results and planned trials need to be viewed in the context of the large mendelian randomization meta-analysis that concluded that CRP itself [13] (unlike the the IL-6 receptor [30]) is not causally-related to coronary heart disease. Indeed, the data from the present study may influence future trial design and lead to careful consideration of the use of hsCRP to select patients at higher cardiovascular risk who may derive greater benefit from targeted treatment.*

3. Given the strong evidence for the pathophysiological effect of a high CRP level, the following statement in the manuscript could be supplemented by citing recent literature: "The striking finding that patients in the highest hsCRP group, but who were troponin-negative, had similar mortality to those who were troponin-positive but had hsCRP<2 mg/L, brings into question whether, in clinical practice, we are missing high-risk patients who may benefit from further investigation and treatment."

Thank you for this suggestion. In reference to our finding we have cited a study evaluating CRP alone and in conjunction with troponin for predicting 14-day mortality in patients with ACS. Elevated CRP at presentation in patients with ACS was correlated with increased 14-day mortality, even in patients with a negative troponin result.

**Page 16, line 27**

*The striking finding that patients in the highest hsCRP group, but who were troponin-negative, had similar mortality to those who were troponin-positive but had hsCRP<2 mg/L, brings into question whether, in clinical practice, we are missing high-risk patients who may benefit from further investigation and treatment [10].*

*10.	Morrow DA, Rifai N, Antman EM, Weiner DL, McCabe CH, Cannon CP, et al. C-reactive protein is a potent predictor of mortality independently of and in combination with troponin T in acute coronary syndromes: a TIMI 11A substudy. Thrombolysis in Myocardial Infarction. Journal of the American College of Cardiology. 1998;31(7):1460-5. Epub 1998/06/17. doi: 10.1016/s0735-1097(98)00136-3. PubMed PMID: 9626820.*

4. Following this, the authors should also introduce CRP not only as end-protein read-out of the IL-6 cascade. They write this in the context of atherosclerosis and baseline CRP levels but should include CRP's pathophysiological role during and after acute incidents.

Thank you for bringing this important concept to our attention. We further discuss the potential role of CRP as a mediator of cardiovascular disease, as well as a marker, in our response to reviewer comment 2, citing the relevant, most up-to-date literature.

5. The statistical methods are well described and mostly appropiate. Among others, the continuous NRI and IDI are used to show the improvement in the prediction model, when hsCRP and troponin positivity are added and p-values are calculated. Continuous NRI and IDI are frequently used, but also prone to error, especially if nested models are used and p-values are calculated (Pepe et al., 2014, Pencina et al., 2015, Burch et al. 2017). The continuous NRI and IDI could be used to describe the improvement of the model, but likelihood-based methods should be used  to calculate p-values (Pepe et al. 2013, Burch et al. 2017).

   Since there are already significant differences in the AUROC for the different models and there is a clear improvement in NRI and IDI, it is very likely, that the likelihood-based calculation will support the improvement of the models.

   Literature:

   Pepe et al. 2013: Testing for improvement in prediction model performance, Stat Med. 2013 Apr 30; 32(9): 1467-1482.
   Pepe et al. 2014: Net Risk Reclassification P Values: Valid or Misleading?, J Natl Cancer Inst. 2014 Apr; 106(4): dju041.
   Pencina et al. 2015: RE: net risk reclassification P Values: valid or misleading?, J Natl Cancer Inst. 2014 Nov 27;107(1):355.
   Burch et al. 2017: Net Reclassification Index and Integrated Discrimination Index Are Not Appropriate for Testing Whether a Biomarker Improves Predictive Performance, Toxicol Sci. 2017 Mar; 156(1): 11-13.

Many thanks for this useful suggestion and for providing references to the relevant literature. Likelihood ratio test P-values have been calculated which confirm the significance of the predictive performance testing using IDI and NRI. The P-values remained significant at <0.001 for all model comparisons. We have highlighted this extra test for statistical significance in the legend for Table 2.

***Table 2: hsCRP risk model discrimination, calibration and reclassification***

*Likelihood ratio test P-values have been calculated which confirm the significance of the predictive performance testing using IDI and NRI. The P-values remained significant at <0.001 for all model comparisons.*
