# Supplementary material for: Mortality risk prediction of high-sensitivity C-reactive protein in suspected acute coronary syndrome: A cohort study
Source: PLoS Med. 2022 Feb 22;19(2):e1003911. doi: 10.1371/journal.pmed.1003911 (PMC8863282; doi:10.1371/journal.pmed.1003911)
Supplement: Attachment — Submitted filename: Response to Reviewer Document.docx [file pmed.1003911.s012.docx]

**Second Response to Reviewer Document**

**NIHR Health Informatics Collaborative
CRP-RISK Study**

Kaura A, et al.

1) In revising the manuscript for further consideration here, please ensure you address the specific points made by each reviewer and the editors. In your rebuttal letter you should indicate your response to the reviewers' and editors' comments and the changes you have made in the manuscript. Please submit a clean version of the paper as the main article file. A version with changes marked must also be uploaded as a marked-up manuscript file.

Thank you for your invitation to further revise our manuscript. We have considered all editor and reviewer comments in detail. A ‘tracked changes’ and ‘clean’ versions of the revised manuscript have been uploaded for further review.

2) Please also check the guidelines for revised papers at http://journals.plos.org/plosmedicine/s/revising-your-manuscript for any that apply to your paper. If you haven't already, we ask that you provide a short, non-technical Author Summary of your research to make findings accessible to a wide audience that includes both scientists and non-scientists. The Author Summary should immediately follow the Abstract in your revised manuscript. This text is subject to editorial change and should be distinct from the scientific abstract.

A short, non-technical Author Summary of our research has been included immediately following the Abstract.

Accepted Changes Manuscript – lines 95 to 116

***Author summary***

***Why was this study done?***

- *C-reactive protein (CRP) is a protein produced by the liver, and released into the blood stream in response to inflammation. A blood test called a high-sensitivity CRP (hsCRP) accurately measures low levels of CRP.*
- *In patients presenting with a suspected heart attack, low levels of hsCRP on admission may prove useful in predicting death beyond using troponin, a marker of heart muscle damage.*

***What did the researchers do and find?***

- *In this study of 102,337 patients with suspected heart attack, the higher the hsCRP level, the higher the risk of death.*
- *This relationship was independent of the troponin level in all patients with suspected heart attack, and was further verified in those who were confirmed to have a heart attack.*

***What do these findings mean?***

- *hsCRP is a clinically meaningful marker of risk of death in addition to troponin in patients with suspected heart attack.*
- *hsCRP has potential utility in selecting patients with heart attack for new treatments which target reducing inflammation.*

3) We ask every co-author listed on the manuscript to fill in a contributing author statement. If any of the co-authors have not filled in the statement, we will remind them to do so when the paper is revised. If all statements are not completed in a timely fashion this could hold up the re-review process. Should there be a problem getting one of your co-authors to fill in a statement we will be in contact. YOU MUST NOT ADD OR REMOVE AUTHORS UNLESS YOU HAVE ALERTED THE EDITOR HANDLING THE MANUSCRIPT TO THE CHANGE AND THEY SPECIFICALLY HAVE AGREED TO IT.

We can confirm that all authors have completed the contributing author statement. The authorship list has not been altered since the original submission.

4) Please ensure that the paper adheres to the PLOS Data Availability Policy (see http://journals.plos.org/plosmedicine/s/data-availability), which requires that all data underlying the study's findings be provided in a repository or as Supporting Information. For data residing with a third party, authors are required to provide instructions with contact information for obtaining the data. PLOS journals do not allow statements supported by "data not shown" or "unpublished results." For such statements, authors must provide supporting data or cite public sources that include it.

Due to data protection and governance restrictions under the NIHR Health Informatics Collaborative data sharing agreement, we are unable to extract or publish patient level data. We have provided a contact for any enquiries.

***Data Availability***

*We are unable to extract or publish patient level data due to data protection and governance restrictions under the NIHR Health Informatics Collaborative data sharing agreement. Any request to access data can be made via imperial.cvhic@nhs.net referring to the title of this paper.*

5) To enhance the reproducibility of your results, we recommend that you deposit your laboratory protocols in protocols.io, where a protocol can be assigned its own identifier (DOI) such that it can be cited independently in the future. Additionally, PLOS ONE offers an option to publish peer-reviewed clinical study protocols. Read more information on sharing protocols at https://plos.org/protocols?utm_medium=editorial-email&utm_source=authorletters&utm_campaign=protocols

The data acquisition and statistical analysis plan are available as S1 Data Acquisition and S1 Analysis Plan, respectively. Sufficient level of detail has been included in the supplementary material to allow reproducibility of our results.

6) Please review your reference list to ensure that it is complete and correct. If you have cited papers that have been retracted, please include the rationale for doing so in the manuscript text, or remove these references and replace them with relevant current references. Any changes to the reference list should be mentioned in the rebuttal letter that accompanies your revised manuscript.

We have reviewed the reference list and confirm that it is both complete and accurate.

7) Please note, when your manuscript is accepted, an uncorrected proof of your manuscript will be published online ahead of the final version, unless you've already opted out via the online submission form. If, for any reason, you do not want an earlier version of your manuscript published online or are unsure if you have already indicated as such, please let the journal staff know immediately at plosmedicine@plos.org.

There are no reservations with the uncorrected proof being published online ahead of the final version.

**Requests from Editors**

1) Please update your line numbering such that it is continuous throughout the document (i.e. does not restart with each new page).

The line numbering for the manuscript has been updated such that it is continuous throughout the document.

2) Please update your full title to ‘Mortality risk prediction of high sensitivity C-reactive protein in suspected acute coronary syndrome: a cohort study’.

The title has been condensed and updated according to the suggestion from the editor.

3) Please trim your abstract as it is currently slightly too long. Key information to include: study design, population and setting, number of participants, years during which the study took place, length of follow up, main outcome measures, quantified main results (with 95% CIs and p values), and important dependent variables adjusted for in the analyses.

Additional methodological detail (e.g. detailed exclusion criteria [lines 14-16], standardisation of troponin levels [lines 17-18], exposure classification [lines 18-19]) can be removed. Additionally, you can remove the content from ‘All analyses on hsCRP…’ to ‘…using the peak troponin level’ in paragraph two of the abstract methods and findings (lines 24-27).

The abstract has been condensed to 361 words. In particular, we have removed any unnecessary detail from the methods section.

Accepted Changes Manuscript – lines 52 to 91

***Background***

*There is limited evidence on the use of high-sensitivity CRP (hsCRP) as a biomarker for selecting patients for advanced cardiovascular therapies in the modern era. The prognostic value of mildly elevated hsCRP beyond troponin in a large real-world cohort of unselected patients presenting with suspected acute coronary syndromes (ACS) is unknown. We evaluated whether a mildly elevated hsCRP (up to 15 mg/L) was associated with mortality risk, beyond troponin level, in patients with suspected ACS.*

***Methods and Findings***

*We conducted a retrospective cohort study based on National Institute for Health Research Health Informatics Collaborative data of 257,948 patients with suspected ACS who had a troponin measured at five cardiac centres in the United Kingdom between 2010 and 2017. Patients were divided into four hsCRP groups (<2, 2-4.9, 5-9.9 and 10-15 mg/L). The main outcome measure was mortality within 3 years of index presentation. The association between hsCRP levels and all-cause mortality was assessed using multivariable Cox regression analysis adjusted for age, sex, haemoglobin, white cell count, platelet count, creatinine and troponin.*

*Following the exclusion criteria, there were 102,337 patients included in the analysis (hsCRP <2 mg/L (n=38,390), 2-4.9 mg/L (n=27,397), 5-9.9 mg/L (n=26,957) and 10-15 mg/L (n=9,593)). On multivariable Cox regression analysis, there was a positive and graded relationship between hsCRP level and mortality at baseline, which remained at three-years (hazard ratio (95% CI) of 1.32 (1.18-1.48) for those with hsCRP 2.0-4.9mg/L, and 1.40 (1.26-1.57), and 2.00 (1.75-2.28) for those with hsCRP 5-9.9 mg/L and 10-15 mg/L, respectively. This relationship was independent of troponin in all suspected ACS patients, and was further verified in those who were confirmed to have an ACS diagnosis by clinical coding. The main limitation of our study is that we did not have data on underlying cause of death, however, the exclusion of those with abnormal WCC or hsCRP levels >15mg/L makes it unlikely that sepsis was a major contributor.*

***Conclusion***

*These multi-centre, real-world data from a large cohort of patients with suspected ACS suggest mildly elevated hsCRP (up to 15 mg/L) may be a clinically meaningful prognostic marker beyond troponin and point to its potential utility in selecting patients for novel treatments targeting inflammation.*

4) Please also condense the ‘limitations’ section of the abstract (lines 10-13) such that it is only a single sentence beginning ‘The main limitations of the study are’, or similar.

The limitations section of the abstract has been condensed.

Accepted Changes Manuscript – lines 78 to 81

***Abstract***

***Methods and Findings***

*The main limitation of our study is that we did not have data on underlying cause of death, however, the exclusion of those with abnormal WCC or hsCRP levels >15mg/L makes it unlikely that sepsis was a major contributor.*

5) Given the observational nature of your study, I suggest updating the first sentence of the abstract conclusions to read ‘These multi-centre, real-world data from a large cohort of patients with suspected ACS suggest mildly elevated hsCRP (up to 15 mg/L) may be a clinically meaningful prognostic marker beyond troponin and point to its potential utility in selecting patients for novel treatments targeting inflammation.’

Many thanks for this useful suggestion. The abstract conclusion has been updated accordingly.

6) The Data Availability Statement (DAS) requires revision. If the data are not freely available, please describe briefly the ethical, legal, or contractual restriction that prevents you from sharing it. Please also include an appropriate contact (web or email address) for inquiries (this cannot be a study author).

Many thanks for bringing this to our attention. Due to data protection and governance restrictions under the NIHR Health Informatics Collaborative data sharing agreement, we are unable to extract or publish patient level data. We have provided a contact for any enquiries.

***Data Availability***

*We are unable to extract or publish patient level data due to data protection and governance restrictions under the NIHR Health Informatics Collaborative data sharing agreement. Any request to access data can be made via imperial.cvhic@nhs.net referring to the title of this paper.*

7) Please update your Author Summary using the standard PLOS Medicine structure. In brief, summaries should comprise of 2-3 single sentence bullet points for each of the three questions. See full guidance here (https://journals.plos.org/plosmedicine/s/revising-your-manuscript) and consult previous PLOS Medicine articles for examples (<https://journals.plos.org/plosmedicine/)>.

The Author Summary has been updated using the standard PLOS Medicine structure.

Accepted Changes Manuscript – lines 95 to 116

***Author summary***

***Why was this study done?***

- *C-reactive protein (CRP) is a protein produced by the liver, and released into the blood stream in response to inflammation. A blood test called a high-sensitivity CRP (hsCRP) accurately measures low levels of CRP.*
- *In patients presenting with a suspected heart attack, low levels of hsCRP on admission may prove useful in predicting death beyond using troponin, a marker of heart muscle damage.*

***What did the researchers do and find?***

- *In this study of 102,337 patients with suspected heart attack, the higher the hsCRP level, the higher the risk of death.*
- *This relationship was independent of the troponin level in all patients with suspected heart attack, and was further verified in those who were confirmed to have a heart attack.*

***What do these findings mean?***

- *hsCRP is a clinically meaningful marker of risk of death in addition to troponin in patients with suspected heart attack.*
- *hsCRP has potential utility in selecting patients with heart attack for new treatments which target reducing inflammation.*

8) Page 8, line 12: Please cite specific items within the supplementary material rather than citing the entire section (e.g. S1 Analysis Plan). See our website for more detailed guidance (<https://journals.plos.org/plosmedicine/s/supporting-information)>.

Many thanks for bringing this to our attention. We have updated the manuscript to now cite specific items within the supplementary material.

Accepted Changes Manuscript – lines 187 to 188

***Methods***

*The data acquisition and statistical analysis plan are available as S1 Data Acquisition and S1 Analysis Plan, respectively.*

9) Please add the following statement, or similar, to the Methods: "This study is reported as per the Strengthening the Reporting of Observational Studies in Epidemiology (STROBE) guideline (S1 Checklist)."

We have added this statement to the end of the Methods section.

Accepted Changes Manuscript – lines 235 to 236

***Methods***

*This study is reported as per the Strengthening the Reporting of Observational Studies in Epidemiology (STROBE) guideline (S1 Checklist).*

10) Did your study have a prospective protocol? Please state this (either way) early in the Methods section.

As highlighted in our response to comment 8, the data acquisition and statistical analysis plan are available as S1 Data Acquisition and S1 Analysis Plan, respectively. This has been stated early in the Methods section.

Accepted Changes Manuscript – lines 187 to 188

***Methods***

*The data acquisition and statistical analysis plan are available as S1 Data Acquisition and S1 Analysis Plan, respectively.*

11) Thank you for providing your prospective analysis plan. Changes in the analysis-- including those made in response to peer review comments-- should be identified as such in the Methods section of the paper, with rationale.

We can confirm that there was no deviation in the statistical analysis plan during conduct of the study or following peer-review. This has been clarified in the methods section.

Accepted Changes Manuscript – lines 187 to 189

***Methods***

*The data acquisition and statistical analysis plan are available as S1 Data Acquisition and S1 Analysis Plan, respectively. There was no deviation in the statistical analysis plan during conduct of the study or following peer-review.*

12) Page 18, line 5: Please remove the citation for Supplementary Figure 2.

The citation for Supplementary Figure 2 has been removed from the Discussion.

13) References: Please ensure all journal names are consistently abbreviated using the abbreviations found in the National Center for Biotechnology Information (NCBI) databases.

We have revised the references to ensure journal names are consistently abbreviated with reference to the NCBI database.

14) Please define the statistical test used to derive the p values in the legend of Table 2.

The area under the receiver operating characteristic curves (AUROC) for each risk model were compared using the DeLong nonparametric approach. The survIDINRI package on R was used to implement the IDI and NRI for comparing risk prediction models. We have added this additional level of detail in the Statistical analysis section. References for both statistical tests are cited in the ‘Data Analysis Plan’. We have also defined both tests in the legend of Table 2.

Accepted Changes Manuscript – lines 271 to 273

***Statistical analysis***

*Discrimination of the different models was assessed by comparing the areas under the receiver operating characteristic curves (AUROC) using the DeLong nonparametric approach.*

Accepted Changes Manuscript – lines 289 to 290

***Statistical analysis***

*The survIDINRI package on R was used to implement the IDI and NRI for comparing risk prediction models.*

Table 2

***Table 2. hsCRP risk model discrimination, calibration and reclassification***

*Discrimination of the different models was assessed by comparing the AUROC using the DeLong nonparametric approach. The survIDINRI package on R was used to implement the IDI and NRI for comparing risk prediction models. Likelihood ratio test P-values have been calculated which confirm the significance of the predictive performance testing using IDI and NRI. The P-values remained significant at <0.001 for all model comparisons.*

15) You allude to statistical testing to determine baseline differences between participants; however no p values are reported in Table 1. Please update Table 1 to include relevant p values and report the statistical test used in the legend.

Apologies for this oversight. Comparisons between hsCRP groups with regard to baseline characteristics were explored by Kruskal-Wallis one-way analysis of variance and χ2 test for trend for continuous and categorical variables, respectively. We have updated the statistical analysis section of the methods with this additional level of detail. The P-values calculated have been reported in Table 1. Unsurprisingly, due to the large patient numbers, small differences in summary estimates between the groups for each variable were statistically significant. A description of the statistical tests used for baseline characteristic comparisons between the hsCRP groups have also been added to the legend for Table 1.

Accepted Changes Manuscript – lines 240 to 242

***Methods***

***Statistical analysis***

*Comparisons between hsCRP groups with regard to baseline characteristics were explored using Kruskal-Wallis one-way analysis of variance test and χ2 test for trend for continuous and categorical variables, respectively.*

Table 1

***Table 1. Baseline clinical characteristics***

*P-values were calculated using Kruskal-Wallis one-way analysis of variance test and χ2 test for trend for continuous and categorical variables, respectively.*

16) Thank you for providing your STROBE checklist. Please replace the page numbers with paragraph numbers per section (e.g. "Methods, paragraph 1"), since the page numbers of the final published paper may be different from the page numbers in the current manuscript.

The page numbers have been replaced with paragraph numbers per section. On reviewing the STROBE checklist, we have added additional detail regarding subgroup analyses to the statistical analysis section. The follow-up time has also been summarised in the results section.

Accepted Changes Manuscript – line 246

***Methods***

***Statistical analysis***

*Subgroup analysis were performed for troponin positivity and ACS diagnosis.*

Accepted Changes Manuscript – line 303

***Results***

***Baseline population characteristics***

*The median follow-up was 3.3 years (interquartile range 1.4-5.1).*

17) The terms gender and sex are not interchangeable (as discussed in http://www.who.int/gender/whatisgender/en/ ); please use the appropriate term.

Apologies for the incorrect use of the terms ‘sex’ and ‘gender’. We have amended the manuscript to ensure that the correct term, ‘sex’, is used consistently throughout the manuscript.

**Comments from Reviewers

Reviewer #1**

We thank the authors for addressing our previous concerns. However, the previous supplementary figure on Kaplan-Meier curves by hsCRP level and ACS diagnosis appears to have been removed; was there any reason for this?

We thank reviewer 1 for their feedback on our revised manuscript. The Kaplan-Meier curves for the four hsCRP groups in a subgroup of patients with ACS is shown in Figure 2C in the main paper. The graph for the non-ACS cohort has been reinserted in the supplementary material (S3 Fig) for interest.

**Reviewer #2**

The authors have addressed my concerns. I have no further comments.

We thank reviewer 2 for evaluating our revised manuscript. We are pleased to hear that the reviewer’s concerns have been addressed.
